# In-depth cross-validation of human and mouse CD4-specific minibodies for noninvasive PET imaging of CD4^+^ cells and response prediction to cancer immunotherapy

**DOI:** 10.7150/thno.95173

**Published:** 2024-08-01

**Authors:** Stefania Pezzana, Simone Blaess, Jule Kortendieck, Nicole Hemmer, Bredi Tako, Claudia Pietura, Lara Ruoff, Simon Riel, Martin Schaller, Irene Gonzalez-Menendez, Leticia Quintanilla-Martinez, Alessandro Mascioni, Argin Aivazian, Ian Wilson, Andreas Maurer, Bernd J. Pichler, Manfred Kneilling, Dominik Sonanini

**Affiliations:** 1 Werner Siemens Imaging Center, Department of Preclinical Imaging and Radiopharmacy, University Hospital Tuebingen, University of Tuebingen, Tuebingen, Germany.; 2 Department of Nuclear Medicine, University Hospital Tuebingen, Eberhard Karls University, Tuebingen, Germany.; 3 Cluster of Excellence iFIT (EXC2180) "Image-Guided and Functionally Instructed Tumor Therapies", University of Tuebingen, Tuebingen, Germany.; 4 Department of Pathology and Neuropathology, University Hospital Tuebingen, University of Tuebingen, Tuebingen, Germany.; 5 ImaginAb, Inglewood, United States of America.; 6 German Cancer Consortium (DKTK) and German Cancer Research Center (DKFZ) partner site Tuebingen, Tuebingen, Germany.; 7 Department of Dermatology, University Hospital Tuebingen, University of Tuebingen, Tuebingen, Germany.; 8 Department of Medical Oncology and Pneumology, University Hospital Tuebingen, University of Tuebingen, Tuebingen, Germany.

## Abstract

Increasing evidence emphasizes the pivotal role of CD4^+^ T cells in orchestrating cancer immunity. Noninvasive *in vivo* imaging of the temporal dynamics of CD4^+^ T cells and their distribution patterns might provide novel insights into their effector and regulator cell functions during cancer immunotherapy (CIT).

**Methods:** We conducted a comparative analysis of ^89^Zr-labeled anti-mouse (m) and anti-human (h) CD4-targeting minibodies (Mbs) for *in vivo* positron emission tomography (PET)/magnetic resonance imaging (MRI) of CD4^+^ T cells in human xenografts, syngeneic tumor-bearing wild-type (WT), and human CD4^+^ knock-in (hCD4-KI) mouse models.

**Results:** Both ^89^Zr-CD4-Mbs yielded high radiolabeling efficiencies of >90%, immunoreactivities of >70%, and specific *in vitro* binding to their target antigens. The specificity of *in vivo* targeting of ^89^Zr-hCD4-Mb was confirmed by PET/MRI, revealing ~4-fold greater ^89^Zr-hCD4-Mb uptake in subcutaneous hCD4^+^ hematopoietic peripheral blood acute lymphoblastic leukemia tumors (HPB-ALL) than in solid hCD4^-^ diffuse histiocytic lymphomas (DHL) and ^89^Zr-mCD4-Mb uptake in hCD4^+^ HPB-ALL tumors. In a comparative cross-validation study in anti-programmed death ligand (αPD-L1)/anti-4-1BB-treated orthotopic PyMT mammary carcinoma-bearing hCD4-KI and WT mice, we detected 2- to 3-fold enhanced species-specific ^89^Zr-hCD4-Mb or ^89^Zr-mCD4-Mb uptake within CD4^+^ cell-enriched secondary lymphatic organs (lymph nodes and spleens). The ^89^Zr-hCD4-Mb uptake in the PyMT tumors was more pronounced in hCD4-KI mice compared to the WT control littermates. Most importantly, MC38 adenocarcinoma-bearing mice treated with a combination of αPD-L1 and anti-lymphocyte-activation gene 3 (αLag-3) antibodies exhibited ~1.4-fold higher ^89^Zr-mCD4-Mb uptake than mice that were not responsive to therapy or sham-treated mice.

**Conclusion:** CD4 PET/MRI enabled monitoring of the CD4^+^ cell distribution in secondary lymphatic organs and the tumor microenvironment, capable of predicting sensitivity to CIT. Our imaging approach will provide deeper insights into the underlying molecular mechanisms of CD4-directed cancer immunotherapies in preclinical mouse models and is applicable for clinical translation.

## Introduction

Many novel cancer immunotherapies have emerged in recent years, reshaping the cancer treatment landscape. These include immune checkpoint inhibitors (ICIs), chimeric antigen receptor T cells, bispecific T-cell engagers, peptide-based vaccines, and oncolytic virotherapies 1-3. With several FDA/EMA-approved monoclonal antibodies (mAbs) targeting programmed death receptor (PD-1) or its ligand (PD-L1), cytotoxic T lymphocyte antigen 4 (CTLA-4), and lymphocyte-activation gene 3 (LAG-3), ICIs are widely used in clinical practice for several tumor types, including melanoma, lung cancer, lymphoma, and renal cell carcinoma 1, 4-6. While these immunotherapies have shown remarkable success in achieving long-term remission even in patients with metastatic and chemotherapy-resistant cancers, their current overall response rates remain unsatisfactory, ranging from 12% to 60% depending on the tumor type and therapeutic combination 1, 6-9. Moreover, currently available genomic or immunohistochemical expression patterns, such as PD-L1 expression, tumor mutational burden, and microsatellite instability, from tumor biopsies exhibit limited predictive value in clinical practice 10-14, underscoring the pressing need for robust biomarker identification that can reliably stratify patients and predict immunotherapy efficacy.

While CD8^+^ cytotoxic T lymphocytes (CTLs) are well established as the primary cell population conveying cytotoxic antitumoral responses, mounting evidence emphasizes the pivotal role of CD4^+^ T helper cells in orchestrating cancer immunity 15, 16. Based on their differentiation, tumor antigen (TA)-specific CD4^+^ T helper cells can exhibit either protumoral or antitumoral functions 17, 18. Interferon-gamma (IFNγ)-producing CD4^+^ T helper (Th1) cells are highly efficient antitumoral players because of their ability to induce polarization into proinflammatory M1 macrophages, cross-prime CTLs, initiate the recruitment of dendritic and natural killer (NK) cells 16, 19-22, or induce tumor senescence 23.

In contrast, TA-specific interleukin (IL)-4-producing CD4^+^ T helper (Th2) cells evolved as a negative prognostic marker within the tumor microenvironment (TME) as they promote tumor growth by enhancing angiogenesis and inhibiting cell-mediated immunity 24-26. CD4^+^ T cells can also acquire a regulatory phenotype (Tregs) essential to downregulate excessive T cell responses and inhibit antitumoral immune responses 27-29.

Besides Th1, Th2 cells, and Tregs, several other CD4^+^ T cell subsets, including IL-9- (Th9), IL-17- (Th17), and IL-22-producing (Th22) T helper cells, have been identified with distinct functions 16, 30, 31. Recent studies have described a TA-specific CD4^+^ T-cell subtype with direct cytotoxic effects 32-34. The CD4 antigen was found to be expressed on subsets of NK cells, monocytes, and macrophages at lower levels and is involved in differentiation, migration, and cytokine expression 35.

The absence of reliable biomarkers for the prediction of efficient antitumoral immune responses upon cancer immunotherapy and the growing interest in harnessing CD4^+^ T cells for their antitumoral potential prompted us to embark on in-depth validation of a minibody-based strategy for tracking endogenous CD4^+^ T-cell dynamics within tumors and lymphatic organs *in vivo* by whole-body immune positron emission tomography (immunoPET). This technique offers a direct and noninvasive imaging approach combining the sensitivity of PET isotopes with the specificity of antibodies applicable for monitoring the expression of cancer and immune cell surface proteins 36, 37.

To date, only few CD4^+^ cell-targeting PET tracers have been validated for use in animal models. Kim *et al.* evaluated a ^89^Zr-labeled human CD4-specific therapeutic antibody ibalizumab and a rhesus F(ab´)_2_ fragment to noninvasively quantify CD4^+^ cells in rhesus macaques 38. Kristensen *et al.* correlated PET uptake of a murine CD4-specific F(ab´)_2_ tracer with *ex vivo* CD4^+^ T-cell tumor infiltration in seven preclinical syngeneic tumor models and monitored infiltration upon αPD-1-based checkpoint inhibitor therapy (CIT)^39^. In addition, Clausen *et al.* applied an identical tracer to longitudinally investigate collagen-induced arthritis mouse models 40. Wu and colleagues developed a biologically inert murine CD4-specific cys-diabody PET tracer (~50 kDa) 41 to monitor the repopulation of CD4^+^ T cells after hematopoietic stem cell transplantation 42 and CD4^+^ T-cell migration into sites of inflammation in DSS-induced experimental colitis 43. Recently, we successfully developed a human CD4-targeting single domain antibody (nanobody)-based immunoPET probe (~15 kDa) to enable CD4^+^ cell tracking in the clinical setting and validated its biological properties and biodistribution in a human-CD4 knock-in mouse model 44. Nagle *et al.* implemented a human-specific CD4 scFv-CH3 fragment (minibody, Mb, ~80 kDa) radiolabeled with ^64^Cu to follow human CD4^+^ T cell migration into a patient-derived glioblastoma 45.

In this study, we radiolabeled anti-mouse and anti-human CD4-targeting Mbs with ^89^Zr using the chelator desferrioxamine (dfo) and extensively validated both immunotracers in xenograft, syngeneic, and hCD4 knock-in (KI) tumor mouse models to assess their specificity and cross-species comparability. Furthermore, we evaluated the potential of these compounds as imaging biomarkers for predicting the efficacy of ICI-based cancer immunotherapies. To our knowledge, this is the first study to analyze human- and mouse-specific immunotracers comparatively. As a cross-species theranostic approach, CD4-PET might be applied to novel immunotherapies in preclinical mouse models and subsequently used in clinical studies for therapy guidance and response evaluation. Thus, noninvasive *in vivo* monitoring of CD4^+^ cells with CD4-specific immunotracers holds the promise of providing valuable insights into the efficacy of cancer immunotherapy and will facilitate a better understanding of T-cell-based immunotherapies and CD4^+^ T-cell-mediated immune responses in immune-mediated inflammatory diseases.

## Results

### ^89^Zr-hCD4-Mbs and ^89^Zr-mCD4-Mbs specifically bind to their target antigens *in vitro*

**Figure 1:**
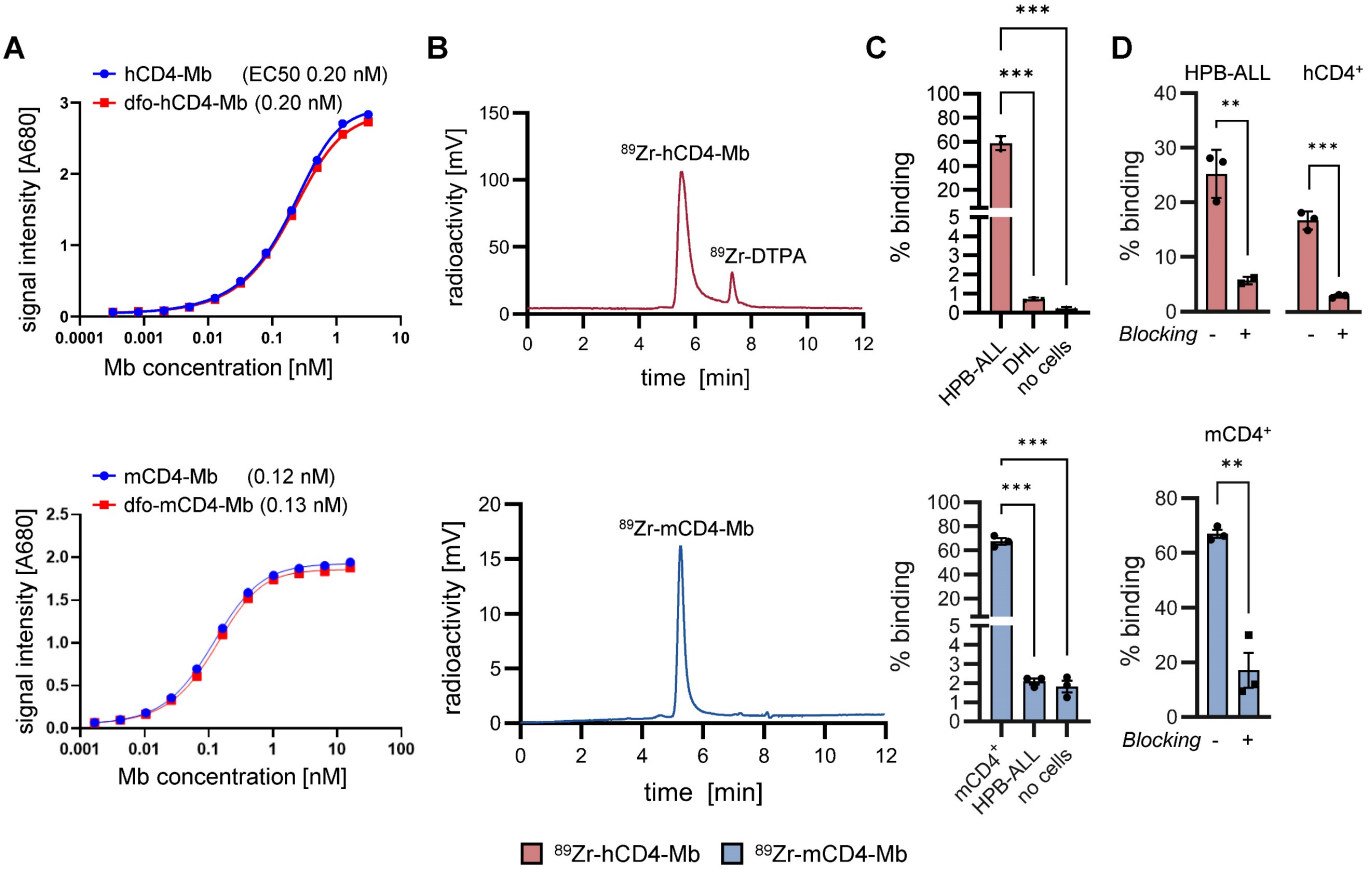
*** In vitro* validation of ^89^Zr-hCD4-Mb and ^89^Zr-mCD4-Mb.** (A) ELISA assay of hCD4-Mb (top) and mCD4-Mb (bottom) with or without dfo conjugation to recombinant human CD4-his or mCD4-his protein. (B) HPLC chromatograms of dfo-h/mCD4-Mb after radiolabeling with ^89^Zr. (C) Total binding of ^89^Zr-h/mCD4-Mb under antigen excess conditions using HPB-ALL, DHL, or freshly isolated mCD4^+^ cells as indicated (40 x 10^6^ cells per well, triplicates). (D) Total binding of ^89^Zr-h/mCD4-Mb to HPB-ALL cells, freshly isolated hCD4^+^ or mCD4^+^ cells as indicated (2 x 10^6^ cells per well, triplicates) with or without blocking with a 100-fold excess of unlabeled h/mCD4-Mb. *P* values were calculated by ordinary one-way and Tukey post-hoc (C) or unpaired t-test for pairwise comparisons (D). The data are presented as mean ± SD. *p < 0.05, **p < 0.01, ***p < 0.001; ****p < 0.0001.

For radiolabeling with ^89^Zr, hCD4- and mCD4-Mbs were conjugated to the radiochelator dfo, which did not impair the affinity in the (sub)nanomolar range as revealed by ELISA (Figure 1A) and flow cytometry (Figure S1A). Subsequent ^89^Zr-radiolabeling yielded >90% radiolabeling efficiency, as determined by HPLC (Figure 1B). Maximum binding assays with increasing numbers of cells (Figure S1B) showed immunoreactive fractions of 78.4% for ^89^Zr-hCD4-Mb using CD4-expressing human HPB-ALL cells and 84.3% for ^89^Zr-mCD4-Mb using murine CD4^+^ T cells. We further confirmed the hCD4-expression on HPB-ALL cells (Figure S1D) and the specific binding of both tracers to the target antigen by species-specific CD4^+^ and CD4^-^ *in vitro* cell assays (Figure 1C, Figure S1E) and through CD4 blocking experiments using a 100-fold excess of unlabeled CD4-Mb. Furthermore, we tested for potential cross-reactivity of both tracers by ELISA with hCD4- or mCD4 using both tracers. Here, neither the hCD4-Mb bound to mouse mCD4, nor the mCD4-Mb to human CD4 (Figure S1C). Most importantly, hCD4-Mb did not induce human PBMC proliferation under coated or soluble conditions (Figure S1E) and remained as stable radioimmunoconjugate over 72 hours in serum (Figure S1F), prerequisite for potential clinical application.

### hCD4-specific *in vivo* binding of ^89^Zr-hCD4-Mb to hCD4^+^ HPB-ALL xenografts in NSG mice

**Figure 2:**
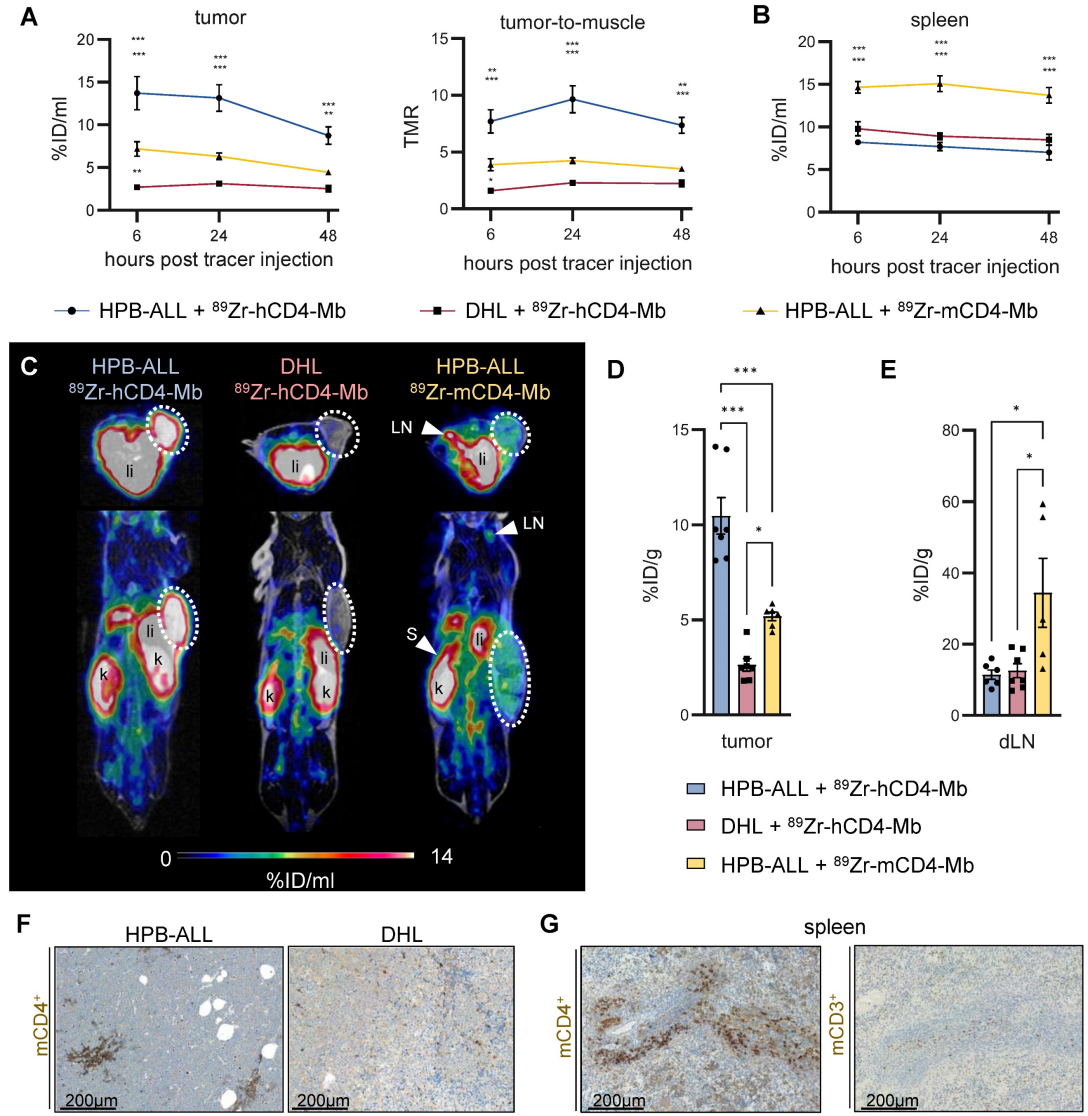
*** In vivo* ^89^Zr-hCD4-Mb and ^89^Zr-mCD4-Mb binding to CD4^+^ cells in immunodeficient NSG mice.** (A) *In vivo* PET uptake quantification and tumor-to-muscle ratio of hCD4^+^ HPB-ALL and hCD4^-^ DHL xenografts 6, 24, and 48 h after ^89^Zr-hCD4-Mb or ^89^Zr-mCD4-Mb injection. (B) *In vivo* PET uptake quantification of the spleen. (C) Representative PET/MR images acquired 48 h after ^89^Zr-hCD4-Mb or ^89^Zr-mCD4-Mb injection. Tumors are highlighted with a white circle. LN: lymph nodes; s: spleen; k: kidney; li: liver. (D) *Ex vivo* biodistribution of hCD4^+^ HPB-ALL and hCD4^-^ DHL tumors and (E) draining lymph nodes (dLNs) measured by γ-counting at 48 h after ^89^Zr-hCD4-Mb or ^89^Zr-mCD4-Mb injection. (F) *Ex vivo* immunohistochemistry (IHC) of endogenous mCD4^+^ cells from HPB-ALL tumors and DHL tumors of NSG mice. (G) *ex vivo* IHC of endogenous mCD4^+^ cells and mCD3^+^ cells from the spleens of NSG mice. *P* values were calculated by two-way ANOVA (A, B) or ordinary one-way ANOVA (D, E) using the Tukey post-hoc test. Data derived from two independent experiments (n = 6-7 per group). *p < 0.05, **p < 0.01, ***p < 0.001.

After we successfully validated radiolabeling and specific *in vitro* binding of ^89^Zr-hCD4-Mb and ^89^Zr-mCD4-Mb to CD4^+^ cells, we examined the *in vivo* PET uptake of ^89^Zr-hCD4-Mb by HPB-ALL leukemia xenografts (which constitutively express the hCD4 antigen) subcutaneously *(s.c.)* implanted into NSG mice. The tumor volumes at the tracer injection time point ranged between 50 and 700 mm^3^ (Figure S2A). We observed a 3.5- to 5-fold increase in the ^89^Zr-hCD4-Mb uptake within hCD4^+^ HPB-ALL tumors compared with hCD4^-^ DHL B-cell lymphomas at 6, 24, and 48 h post tracer injection (Figure 2A, 2C-D; Figure S2C). Interestingly, the ^89^Zr-mCD4-Mb uptake in hCD4^+^ HPB-ALL tumors, which served as another control group, was slightly higher than the ^89^Zr-hCD4-Mb uptake in CD4^-^ DHL tumors (Figure 2A, 2C - D; Figure S2C), indicating mCD4^+^ cell infiltration in T-cell-deficient NSG mice. We detected significantly higher ^89^Zr-mCD4-Mb uptake within the spleen (Figure 2B; Figure S2B,* in vivo*) and in the lymph nodes (Figure 2E,* ex vivo* biodistribution) compared to both groups injected with ^89^Zr-hCD4-Mb. Immunohistochemistry (IHC) of the tumors and spleens confirmed the presence of a limited number of mCD4^+^ cells, presumably CD4^+^ myeloid cells, based on their nuclear morphology and absence of CD3^+^ cells in the spleen (Figure 2F-G). As expected from the Mb-based tracer, most of both ^89^Zr-CD4-Mbs cleared from the blood within 48 h with minimal residual uptake in the muscles, lungs, and hearts of all groups (Figure S2C-D). Furthermore, ^89^Zr-mCD4-Mb exhibited a higher kidney uptake and lower liver uptake than ^89^Zr-hCD4-Mb at the 48-h *ex vivo* biodistribution time point (Figure S2E), presumably attributed to different excretion kinetics of the radioimmunoconjugates.

In conclusion, this experiment confirmed the specific *in vivo* binding of ^89^Zr-hCD4-Mb to hCD4^+^ cells and the presence of limited densities of mCD4^+^ cells ^89^Zr-hCD4-Mb in the tumor tissue and lymphatic organs of NSG mice.

### Whole-body tracking of endogenous CD4^+^ immune cells in ICI-treated human CD4 knock-in (KI) and wild-type (WT) syngeneic orthotopic breast cancer mouse models

**Figure 3:**
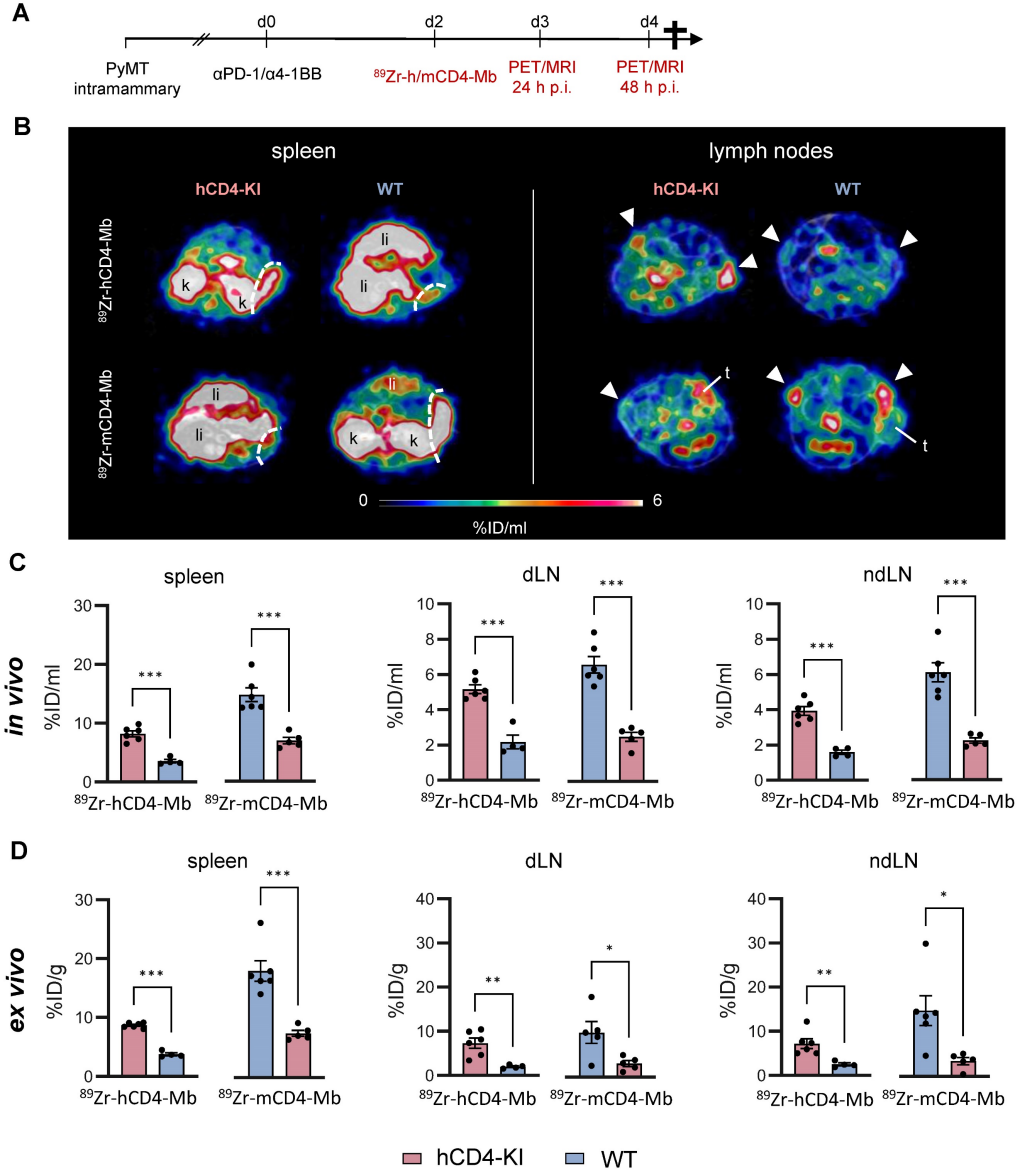
*** In vivo* cross-validation of ^89^Zr-h/mCD4-Mb to visualize endogenous CD4^+^ cells in lymphatic organs.** (A) Treatment and PET imaging schedule. hCD4-KI and WT animals were orthotopically inoculated with 0.5 x10^6^ PyMT cells in the 4^th^ mammary fat pad and treated with a combination of the αPD-1/α4-1BB mAbs (200 μg/50 μg per mouse). PET/MRI was performed 3 and 4 days after treatment onset (24 h and 48 h after *i.v.* injection of ^89^Zr-h/mCD4-Mb). (B) Representative PET/MR images of the spleen (left, separated from the kidney/liver derive uptake by a white broken line) and lymph nodes (right, white triangle) acquired 48 h post-tracer injection. k: kidney; li: liver; t: tumor. (C) *In vivo* quantification of human and murine ^89^Zr-CD4-Mb uptake in lymphatic organs (spleen, draining lymph node (dLN), and contralateral non-draining lymph node (ndLN)) 48 h post tracer injection. (D)* Ex vivo* quantification of ^89^Zr-h/mCD4-Mb uptake in lymphatic organs 48 h post tracer injection. *P* values were calculated by an unpaired t-test. Data derived from two independent experiments (n = 4-6 per group). *p < 0.05, **p < 0.01, ***p < 0.001.

We orthotopically implanted mammary PyMT tumors into wild-type (WT) and hCD4 antigen knock-in (hCD4-KI) mice to further explore endogenous CD4^+^ cells in the tumor, spleen, draining and nondraining lymph nodes (dLNs and ndLNs). All experimental mice were subjected to combined αPD-1/α4-1BB immunotherapy two days before injecting ^89^Zr-hCD4-Mb or ^89^Zr-mCD4-Mb to increase the CD4^+^ T cell density of established tumors (Figure 3A, Figure S4A).

We detected 2-3-fold enhanced ^89^Zr-hCD4-Mb uptake in the spleens and lymph nodes of PyMT tumor-bearing hCD4-KI mice compared with WT mice (Figure 3B, C; Figure S3A-B, S4B). Likewise, we found a similar 2-3-fold increased PET uptake with ^89^Zr-mCD4-Mb in the lymphatic organs of PyMT tumor-bearing mCD4^+^ WT mice compared to non-species-specific hCD4-KI mice (Figure 3B, C; Figure S3A-B, S4B). Interestingly, the absolute ^89^Zr-mCD4-Mb PET uptake was higher in the lymphatic organs of species-specific and non-species-specific groups than the ^89^Zr-mCD4-Mb PET uptake (Figure 3B-D). Together with the different excretion kinetics within the NSG mouse models (Figure S2E), these data suggested a slightly different biodistribution between ^89^Zr-mCD4-Mb and ^89^Zr-hCD4-Mb in a target-unrelated manner. Also, the *ex vivo* biodistribution analysis of lymphatic organs confirmed our *in vivo* ^89^Zr-hCD4-Mb-PET/MRI results (Figure 3D). In addition, we detected a similar αCD4 PET uptake in the tumor-draining and non-draining lymph nodes (Figure 3B-D).

**Figure 4:**
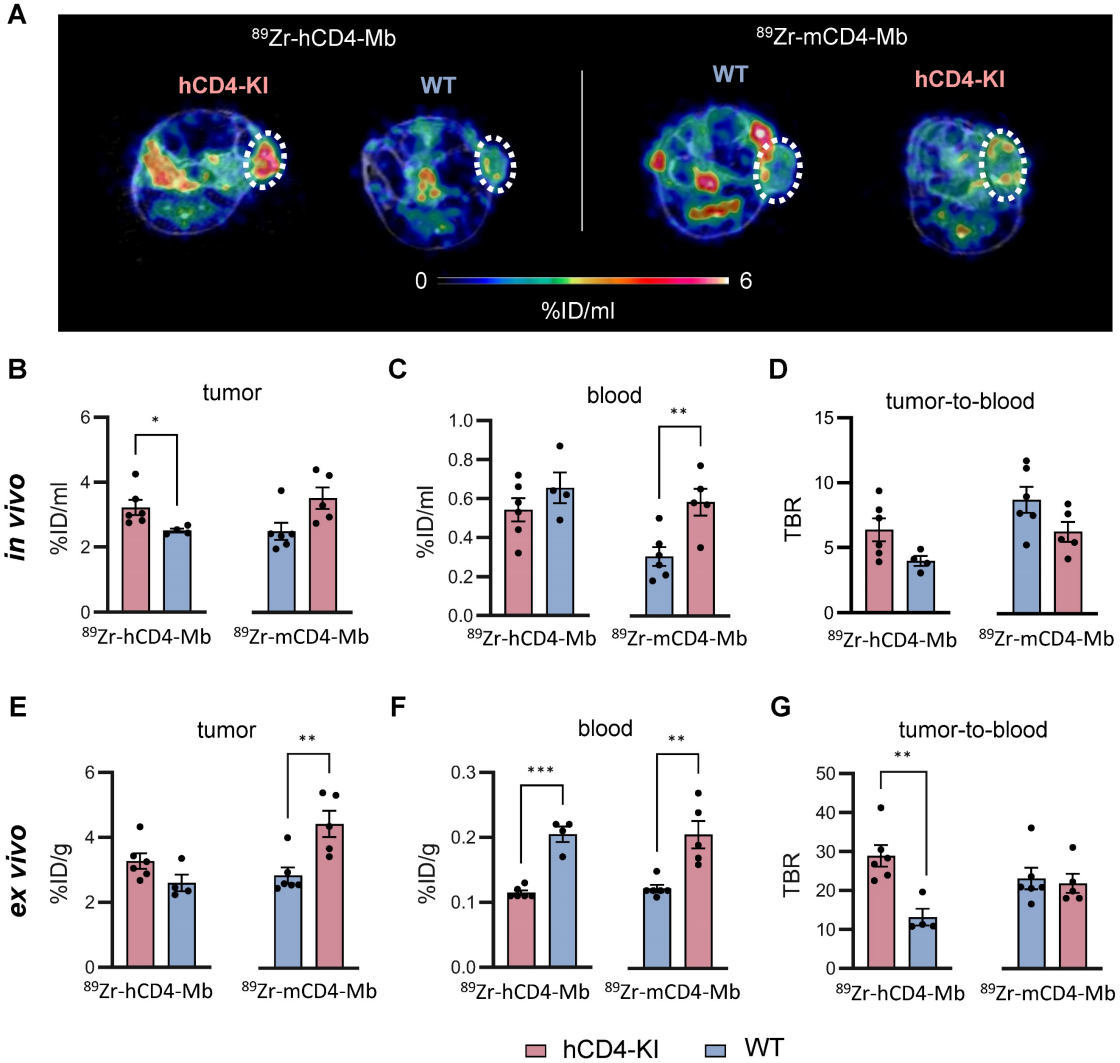
** Noninvasive visualization of tumor-infiltrating endogenous CD4^+^ cells in hCD4-KI and WT PyMT mammary tumor-bearing mice.** (A) Representative *in vivo* PET/MR images of PyMT mammary tumors acquired 48 h post-^89^Zr-CD4-Mbs i.v. injection (day 4 post-αPD-1/α4-1BB mAb injection). Tumors are highlighted by white circles. (B) *In vivo* quantification of ^89^Zr-h/mCD4-Mb uptake in PyMT tumors and (C) blood and (D) the tumor-to-blood ratio in hCD4-KI and WT mice 48 h post tracer injection. (E) *Ex vivo* quantification of ^89^Zr-h/mCD4-Mb uptake in PyMT tumors and (F) blood as well as (G) the tumor-to-blood ratio for hCD4-KI and WT mice at 48 h post tracer injection measured by γ-counting. *P* values were calculated by unpaired t-test. Data are derived from two independent experiments (n = 4-6 per group). *p < 0.05, **p < 0.01, ***p < 0.001.

Next, we focused on ^89^Zr-hCD4-Mb and ^89^Zr-mCD4-Mb uptake within the TME and detected significantly enhanced ^89^Zr-hCD4-Mb uptake in αPD-1/α4-1BB-treated PyMT tumor-bearing hCD4-KI mice when compared to WT mice (3.22 ± 0.23 vs. 2.51 ± 0.06 %ID/ml, Figure 4A, B). Surprisingly, ^89^Zr-mCD4-Mb PyMT tumor uptake was even higher in hCD4-KI mice than in target-specific WT mice expressing mCD4^+^ (Figure 4A, B). To further elaborate on whether target-specific tracer consumption effects in the lymphatic organs occurring only in species-specific mice were responsible for these unexpected observations, we analyzed the *in vivo* blood uptake in all experimental mice. We found a 1.9-fold lower ^89^Zr-mCD4-Mb PET uptake in the blood of WT mice than hCD4-KI control mice (Figure 4C), resulting in a pronounced species-specific ^89^Zr-mCD4-Mb PET uptake when corrected for blood background (8.76 ± 0.99 vs. 6.30 ± 0.77, non-significant, Figure 4D). These findings also applied to the ^89^Zr-hCD4-Mb to a lesser extent (Figure 4C-D) and could be confirmed by *ex vivo* biodistribution analyses of the tumor and blood (Figure 4E-G).

Despite the limitations of knock-in/out mouse models in the absolute quantification of antibody-based immunotracers, we could quantitatively measure increased and comparable PET uptake patterns of the lymphatic organs and the TME in a syngeneic orthotopic tumor mouse model for ^89^Zr-hCD4-Mb and ^89^Zr-mCD4-Mb.

### Intratumoral ^89^Zr-mCD4-Mb uptake as a response indicator for combined immunotherapy

**Figure 5:**
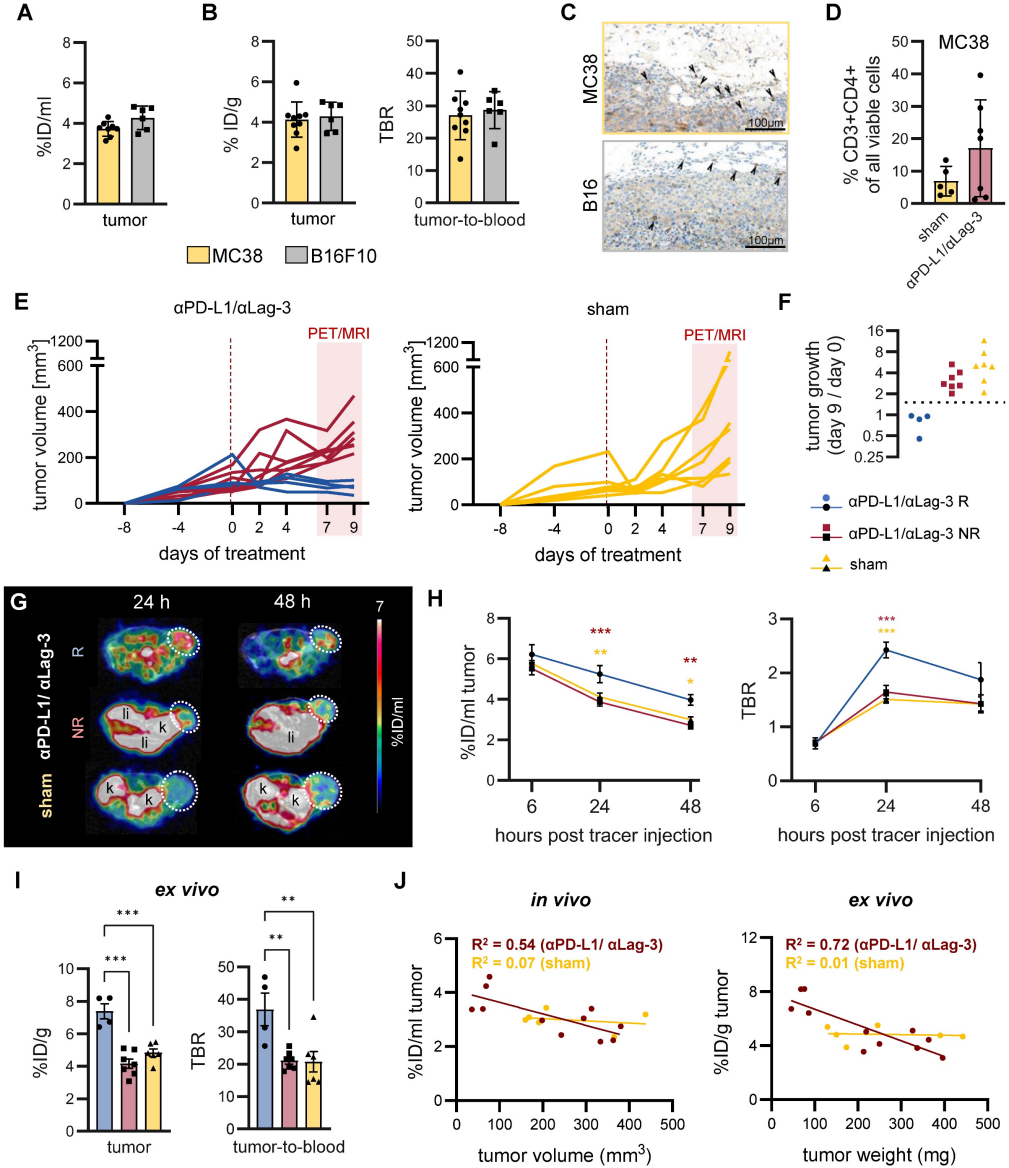
***In vivo* ^89^Zr-mCD4-Mb PET for monitoring endogenous CD4^+^ cell dynamics in response to ICI therapy.** (A) *In vivo* tumor uptake quantification of endogenous mCD4^+^ cells in subcutaneous MC38 (n = 9) and B16F10 (n = 6) tumors 48 h after ^89^Zr-mCD4-Mb tracer injection. (B) *Ex vivo* γ-counting of the tumor and *ex vivo* tumor-to-blood ratio (TBR) 48 h after ^89^Zr-mCD4-Mb injection. (C) *Ex vivo* mCD4 IHC of MC38 and B16 tumors. Black arrows indicate mCD4^+^ cells. (D) Fraction of intratumoral CD4^+^ cells of all cells from αPD-L1/αLag3-treated (n = 7) and sham-treated animals (n = 5) by flow cytometry. (E) MC38 tumor volumes (mm^3^) of αPD-L1/αLag-3-treated or sham-treated mice. The red rectangle indicates the PET/MR acquisition period at day 7 (6 h post tracer injection), day 8 (24 h), and day 9 (48 h). (F) Tumor growth ratio on day 9 vs. day 0 (baseline). Based on the tumor growth ratio (tumor volume at day 9 / tumor volume at day 0), mice were considered responsive (<1.5, R, blue) or non-responsive to treatment (>1.5, NR, red). (G) Representative *in vivo* PET/MR images 24 h and 48 h after ^89^Zr-mCD4-Mb tracer injection. Tumors are highlighted with a white circle. k: kidney; li: liver. (H) *In vivo* ^89^Zr-mCD4-Mb MC38 tumor uptake and tumor-to-blood ratio (TBR) were quantified at 6 h, 24 h, and 48 h post tracer injection. (I) *Ex vivo* quantification of ^89^Zr-mCD4-Mb uptake in MC38 tumors and tumor-to-blood ratio measured by γ-counting. (J) Correlation of *in vivo* (left) and *ex vivo* (right) ^89^Zr-mCD4-Mb uptake with the corresponding tumor volume and tumor weight, respectively, at 48 h after ^89^Zr-mCD4-Mb tracer injection. Data are derived from two independent experiments (αPD-L1/αLag3: n = 11, sham: n = 6; four mice were excluded from the study because of ulcerated tumors). *p < 0.05, **p < 0.01, ***p < 0.001.

Finally, we aimed to evaluate the sensitivity of ^89^Zr-mCD4-Mb PET for monitoring moderate alterations in CD4^+^ T cell density within the TME before and during ICI treatment and its correlation with the treatment sensitivity. We injected ^89^Zr-mCD4-Mb into CIT-responsive MC38 adenocarcinoma-bearing mice and into CIT-resistant B16F10 melanoma-bearing mice when the tumors reached a diameter of 8-10 mm (mean volume: ~300 mm^3^, Figure S54A) before therapy initiation. The noninvasive *in vivo* ^89^Zr-mCD4-Mb PET/MRI studies and subsequent *ex vivo* analyses revealed no differences in ^89^Zr-mCD4-Mb tumor uptake within the TME of MC38 and B16F10 tumors at baseline (Figure 5A, B). IHC confirmed the presence of scattered CD4^+^ T cells in both tumors, mainly located at the tumor periphery (Figure 5C).

Next, only MC38 tumor-bearing mice were subjected to combined αPD-L1/αLag-3 ICI therapy. Interestingly, 4 out of 7 ICI-treated mice yielded a 2.5 to 5-times increased CD4^+^ T-cell density within the MC38 TME compared to the sham-treated controls 9 days post αPD-L1/αLag-3 therapy initiation, suggesting therapy-induced and/or response-associated accumulation of CD4^+^ T cells (Figure 5D). Subsequently, we monitored the presence of CD4^+^ cells by ^89^Zr-mCD4-Mb-PET/MRI on days 7, 8, and 9 after initiating CIT (Figure 5E). αPD-L1/αLag-3-treated MC38 tumor-bearing mice were classified as responders or non-responders based on tumor growth (tumor volume on day 9 / tumor volume on day 0 of therapy) (Figure 5E-F). Strikingly, we observed ~1.4-fold higher ^89^Zr-mCD4-Mb uptake in responsive MC38 tumors from experimental mice 24 h (5.23 ± 0.42 %ID/ml) and 48 h (3.97 ± 0.27 %ID/ml) after tracer injection than in non-responsive MC38 tumors (24 h %ID/ml: 3.87 ± 0.20; 48 h %ID/ml: 2.72 ± 0.17) and ~1.3-fold higher intratumoral uptake than in sham-treated experimental mice (24 h %ID/ml: 4.12 ± 0.20; 48 h %ID/ml: 3.00 ± 0.14; Figure 5G-H; Figure S5C).

Consistent with our *in vivo* results, *ex vivo* organ biodistribution analysis of the tumors, muscle, and blood revealed increased ^89^Zr-mCD4-Mb accumulation in the TME and significantly higher tumor-to-blood and tumor-to-muscle ratios in mice responding to ICI non-responsive MC38 tumors or tumors from sham-treated experimental mice (Figure 5I; Figure S5D). Besides, there were no differences in the *ex vivo* biodistribution of several organs of interest, such as the spleen, draining lymph nodes, liver, kidney, lung, or blood, between all experimental groups (Figure S5D).

To exclude that the differences in tumor volumes between responding and non-responding animals affect the tracer uptake by biological (e.g. tumor penetration and accessibility) or physical (e.g. partial volume effects of the PET detectors) effects, we correlated the *in vivo* tracer uptake with the tumor volumes and the *ex vivo* tracer uptake with the tumor weight (Figure 5J). We observed a homogeneous distribution of the uptake values in the sham-treated group (not influenced by therapy-induced tumor growth inhibition) irrespective of the tumor size. Furthermore, comparable linear regressions and R^2^-values between *in vivo* and *ex vivo* analyses indicated that the tracer signals were influenced by partial volume or other physical effects. Noteworthy, flow cytometry of the TME revealed no relevant fraction of CD4^+^ cells when gated for CD45^+^CD3^-^ myeloid cells (Figure S5B).

### ICI response-associated infiltration of CD4^+^ T cells into TME

**Figure 6:**
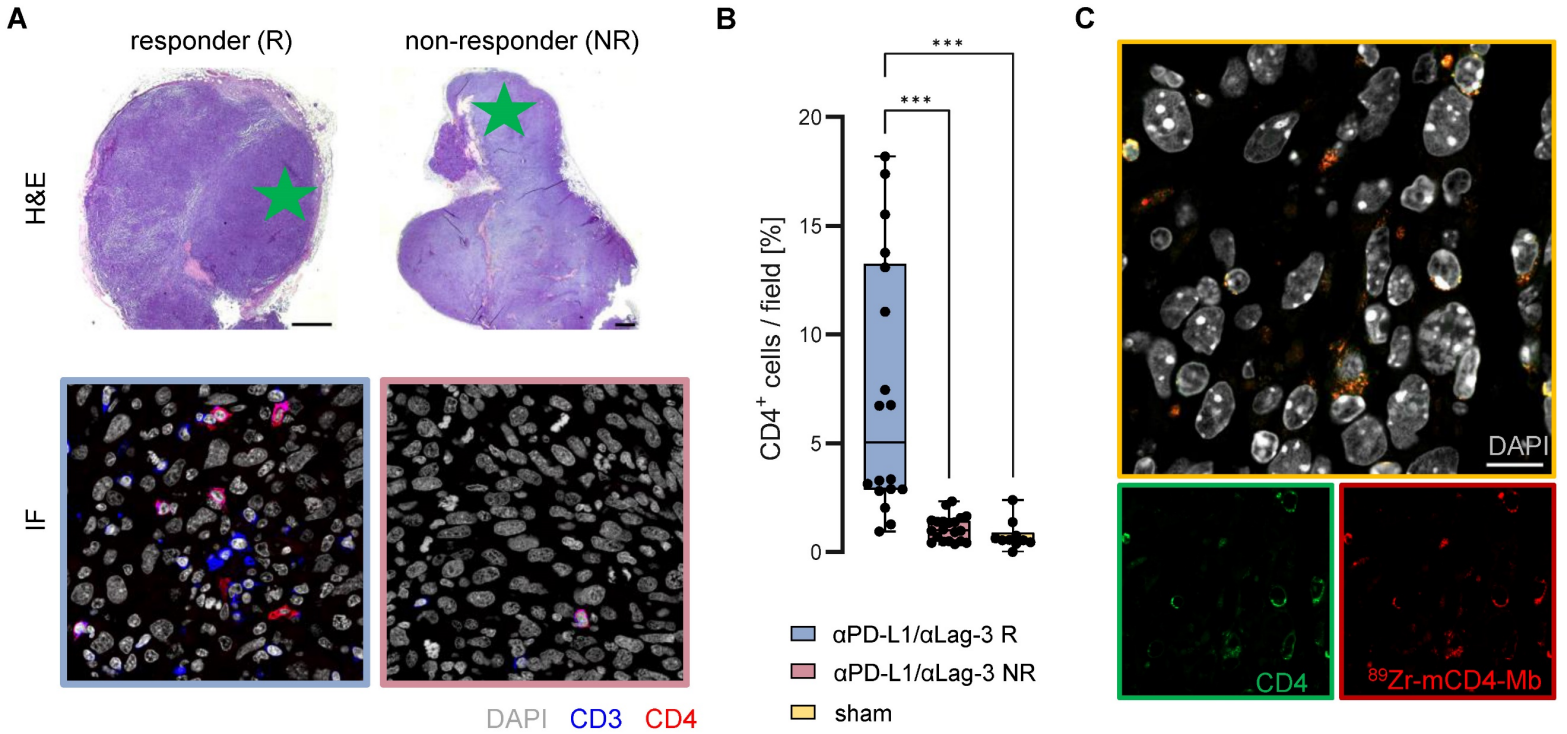
***Ex vivo* validation of ^89^Zr-mCD4-Mb uptake.** (A) *Ex vivo* H&E staining of MC38 tumors from αPD-L1/αLag3-treated mice and representative immunofluorescence (IF) of CD3^+^ and CD4^+^ cells (field marked by a green star). Scale bars: 1000 µm H&E, 20 µm IF. (B) Quantification of % intratumoral CD4^+^ cells of all cells per field (5 fields per tumor) from αPD-L1/αLag3 responders (R, n = 4), non-responders (NR, n = 4) and sham-treated animals (n = 2). (C) Colocalization of intratumoral CD4 protein expression and ^89^Zr-mCD4-Mb by secondary IF staining. Scale bar: 10 µm. *p < 0.05, **p < 0.01, ***p < 0.001.

To better understand the increased ^89^Zr-mCD4-Mb uptake within the TME and the intra-tumoral CD4^+^ T cell distribution of therapy responders, we performed immunofluorescence analyses of the MC38 tumors on day 9 of αPD-L1/αLag-3 therapy after the 48 h PET imaging time point. Representative high-resolution images of the tumor margin and the tumor core (Figure 6A) and CD4^+^ cell quantification (Figure 6B) revealed about 7-fold increased infiltration of CD3^+^CD4^+^ T cells into the TME of responsive MC38 tumors, whereas no CD3^+^CD4^+^ T cells could be identified in nonresponsive MC38 tumors or tumors from sham-treated mice (Figure 6A, B). Most importantly, we were able to colocalize the ^89^Zr-mCD4-Mb distribution to endogenous tumor-infiltrating CD4^+^ cells by secondary staining of the Mb, highlighting the tracer-specific targeting of CD4^+^ cells within the TME at the cellular level (Figure 6C).

## Discussion

Given the inadequate immunotherapy response rates and the urgent need for reliable biomarkers for therapy stratification, immunoPET approaches have been applied clinically to visualize intratumoral CTLs 46, 47 or TME-associated immune checkpoints in cancer patients 48-50. A pioneering example of the most advanced immunotracer in clinical development was the CD8-targeting PET tracer ^89^Zr-crefmirlimab berdoxam 51, 52. This Mb-based radioimmunoconjugate, currently under evaluation in phase II clinical study and in many pharma- and investigator-initiated trials, demonstrated remarkable capability to assess the therapeutic responses to ICIs and other immunotherapies within the first 3-6 weeks of treatment 47, 51, 52. Due to their lower molecular weight and reduced Fc receptor-mediated interaction compared to full-length antibodies, Mbs have superior molecular imaging characteristics, such as faster tissue penetration, blood clearance, and reduced immunogenicity 47, 51-54. While PD-(L)1-directed ICI therapies, as a standard-of-care treatment for several tumor types, critically depend on CTL-mediated immune responses 55-57, emerging ICI combinations, including CTLA-4, Lag-3, or OX40-targeting mAbs, along with innovative tumor vaccination approaches, emphasize the increasing focus on reinvigorating antitumoral CD4^+^ T-cell activity 31, 56-59.

The lack of clinically translatable human-specific CD4-targeting immunoPET tracers and the promising clinical development of ^89^Zr-crefmirlimab berdoxam motivated us to develop Mb-based PET tracers to monitor endogenous CD4^+^ cell dynamics in the TME and lymphatic organs. First, we verified the *in vitro* and *in vivo* target specificity of ^89^Zr-hCD4-Mb and ^89^Zr-mCD4-Mb. We demonstrated an ~80-fold higher *in vitro* binding of the ^89^Zr-hCD4-Mb in the hCD4^+^ HBP-ALL cells than in the hCD4- DHL B-cell-lymphoma cells while *in vivo* accumulation of s.c. injected cells was only ~4-fold higher. In this context, *in vivo* biodistribution effects, permeability, and unspecific accumulation have to be considered that impair tracer accessibility and specificity, particularly in *s.c.* injected tumors.

We also monitored the presence of endogenous CD4^+^ cells in immune cell-enriched lymphatic organs, such as the spleen and lymph nodes, and in the TME of tumor-bearing WT and hCD4-KI mice. Interestingly, even low numbers of endogenous mCD4^+^ cells present in immunodeficient NSG mice 60-62 or in the TME of MC38 tumors could be differentiated by our ^89^Zr-CD4-Mb immunoPET approach (Figure 2F), highlighting its superior sensitivity. As for visualizing tumor-infiltrating lymphocytes, enhanced tracer accumulation in the lymphatic organs may be considered an antigen sink, resulting in reduced amounts of radiotracer available to bind to target cells at the tumor site 39, 63. This was expected for larger antibody-based molecules and can explain the limitations of head-to-head comparisons with considerable differences in the ^89^Zr-CD4-Mb blood biodistribution using knock-out/knock-in mouse models (Figure 4). To avoid this, immune cell-rich organs could be saturated by administering an excess of an unlabeled antibody targeting the same antigen or a higher dose of the immunoPET tracer to increase the availability of the probe at the tumor site 39, 63, 64. Therefore, optimizing the dose may be essential for obtaining a sufficient imaging signal, while pre-dosing strategies pose a risk of perturbing the binding of tracers to intratumoral immune cells 39.

Besides the target abundance, several factors, including microvascular density, vascular permeability, stromal content, intratumoral pressure, and diffusion, influence tumor accumulation of immunoPET tracers 65. In our study, correcting for ^89^Zr-mCD4-Mb and ^89^Zr-hCD4-Mb uptake in the TME with the blood uptake enabled us to detect a species-specific Mb signal in experimental PyMT tumor-bearing WT and hCD4-KI mice 39. One of the main concerns in developing new immunotracers is the effects on targeted cells, such as unintentional immune cell activation or the inhibition of immune cell effector functioning. Our study revealed no impact of hCD4-Mb on the proliferation of human PBMCs *in vitro*. Similarly, the injection of ^64^Cu-labeled hCD4-Mb by Nagle *et al.* did not result in detectable depletion or alterations in the proliferation or polarization of hCD4^+^ cells in a humanized glioblastoma model 45. These findings hold particular significance for translational studies on tracking CD4^+^ cells by hCD4-Mbs in patients. Also, Freise *et al.* could not detect alterations in CD3^+^ T-cell or CD45^+^CD4^+^ cell compartments in the spleen, lymph nodes, thymus, or blood using a CD4-targeting ^89^Zr-radiolabeled cys-diabody despite a mild reduction of CD4-expressing immune cells 41. Interestingly, the investigators reported a dose-dependent downregulation of membranous CD4 expression. Notably, decreased membranous CD4 expression has been reported on activated immune cells 66, 67. Nevertheless, Freise *et al.* could not identify a CD4-cys-diabody-induced activation of CD4-expressing immune cells.

Our study did not show differences between ICI-responsive MC38 adenocarcinomas (“hot tumors”) and ICI-resistant B16F10 melanomas (“cold tumors”) at baseline by ^89^Zr-mCD4-Mb immunoPET. The TME of immunogenic MC38 tumors was characterized by myeloid cells and very few CD4^+^ cells (Figure 5C) 68, 69. Also, Kjaer *et al*, who investigated the ^89^Zr-mCD4-F(ab´)2 fragment in seven preclinical syngeneic tumor models (MC38, CT26, B16F10, 4T1, P815, RenCa, Sa1N), could not show differences in the CD4-derived PET uptake between MC38 and B16F10 tumors. Nevertheless, the group demonstrated a correlation between PET uptake, *ex vivo* CD4^+^ cells densities, and response to aPD1 ICI therapy in some tumor models, which was not the case using a CD8-directed ^89^Zr-mCD8-F(ab´)2 39.

Given the fact that CD4^+^ cells represent a very heterogenous group with either protumoral or antitumoral function, we investigated whether ^89^Zr-mCD4-Mb PET could assess early ICI response or resistance. Strikingly, we could discriminate MC38 tumor-bearing mice that were responsive (higher PET uptake) or nonresponsive (lower PET uptake) to αPD-L1/αLag-3 immunotherapy 7 days after therapy initiation. This finding was consistent with our IF analyses, demonstrating significant CD4^+^ T cell infiltration into the TME in therapy responders (Figure 6A-C) 70, 71. In conclusion, our study presented a comprehensive preclinical cross-validation of ^89^Zr-CD4-Mb as a precision tool for noninvasive monitoring and visualizing endogenous CD4^+^ cells throughout the body.

Beyond oncological purposes, Mascio *et al.* performed valuable research on the whole-body PET visualization of CD4^+^ T cells in non-human primates by using dynamic PET data advanced mathematic modeling approaches. They applied the therapeutic anti-human CD4 full-length antibody ibalizumab and rhesus-specific F(ab')_2_ antibody fragments to immunocompetent and Simian Immunodeficiency Virus-infected rhesus macaques. The overall capability of both radioimmunoconjugates to differentiate increased CD4^+^ T-cell densities of the spleen and lymph nodes appeared comparable to our results. However, the limitations of therapeutic antibodies for diagnostic imaging, observed dose- and blood pool-dependent uptake kinetics, and the use of a primarized F(ab')_2_ hinder their clinical translatability.

Considering the already demonstrated clinical success of ^89^Zr-crefmirlimab berdoxam in targeting human CD8^+^ cells 51, 52, our Mb-based imaging approach offers a remarkable balance between high target specificity and low toxicity due to its rapid biodistribution and clearance from blood. Based on increasing histology-driven evidence from human tumor tissues suggesting CD4^+^ cells as critical biomarkers to determine therapy efficacy in oncolytic virus 72, CAR T cells 73, and immune checkpoint inhibitor therapy 74-79, ^89^Zr-CD4-Mb immunoPET holds great promise for clinical translation as a valuable tool for the noninvasive monitoring of CD4^+^ cells, and the patient-individualized prediction of cancer immunotherapy outcomes.

## Methods

### Minibody production

Single-chain variable fragment (scFv) sequences were derived from anti-human-specific mAb MAX16H5 and anti-mouse mAb YTA3.1.2. Identical to the clinically established CD8-directed Mb PET tracer 89Zr-crefmirlimab berdoxam, these binding domains were fused with a human Fc-CH3 domain, to generate the hCD4-Mb (IAB41M1-3) or mCD4-Mb (IAB46M2-18), respectively, with a final molecular weight of ~80 kDa. Mbs were produced in transiently transfected Expi293™ cells (ThermoFisher Scientific). The purified proteins were conjugated via coupling to primary amines with parabenzoylisothiocyanate-activated deferoxamine (dfo), yielding chelator-to-minibody ratios of 2.45 (hCD4-Mb) and 1.65 (mCD4-Mb), respectively.

### Enzyme-linked Immunosorbent Assay (ELISA)

Recombinant hCD4-His or mCD4-His (both Sino Biologicals) were diluted to 2 μg/mL in the carbonate bicarbonate buffer (Sigma), and 100 μL were added to each well of a flat-bottom plate and incubated at 2-8**°**C overnight. The following day, the plate was washed 3x with Phosphate-Buffered Saline with Tween (PBST 0.05%) and blocked for 1 h at room temperature with PBS containing 1% bovine serum albumin (BSA) (Sigma). The plate was washed 3x with PBST (0.05%). A serial dilution was prepared by diluting the sample 1:2.5 to achieve a standard curve starting from 16 nM for both hCD4-Mb and mCD4-Mb; 100 μL of the sample was added to each well of the test plate and incubated for 1 h at room temperature. The plate was washed 3x with PBST (0.05%). Mouse anti-human IgG Fc-HRP detection antibody (Southern Biotech) was diluted 1:20,000 in assay buffer, and 100 μL was added to each well of the test plate and incubated for 1 h at room temperature. Subsequently, the plate was washed 3x with PBST (0.05%), 100 μL of TMB substrate (BioFx) was added to each well of the test plate and allowed to develop for 10-20 min at room temperature. The reaction was terminated by adding 100 μL of stop reagent (BioFx). The plate was read at 650 nm using a BioTek Synergy II plate reader.

### Human PBMC proliferation assay

The antibodies were coated on plates in triplicate at 40 μg/mL in PBS at +4°C overnight. PBMCs were thawed and incubated in Human Serum AB according to the RESTORE protocol before use and plated at 100,000 cells/well (100 mL total). For the soluble assay format, antibodies were added at a concentration of 65 nM in triplicate to the plate coated with PBMCs. The plates were incubated at 37°C and 5% CO_2_ for 5 days, after which the CTG substrate was added, and the plates were placed on a shaker for 5 min. The luminescence of the plates was subsequently read on a plate reader to determine the extent of proliferation.

### Radiolabeling

The dfo-conjugated hCD4-Mb and mCD4-Mb were radiolabeled with 200 MBq Zr-89 oxalate (Perkin Elmer) per mg protein. Briefly, the desired activity was neutralized with a 0.45-fold volume of 2 M sodium carbonate and buffered with a 5-fold volume of 0.5 M ammonium acetate to achieve a pH of 6.5-7.0. After adding the protein, labeling was allowed to proceed for 40 minutes at 25°C, after which the reaction was quenched by adding DTPA (0.2% solution, 40 µL per mg protein) and incubated for an additional 10 min. Incorporation of the radioisotope was confirmed by iTLC analysis (≥95% radiochemical purity (RCP); iTLC-SG (Agilent), mobile phase 10 mM EDTA). The identity of the elution profile with the original protein and radiolabeling of the protein peak was confirmed by high-performance size exclusion chromatography (HPSEC) (BioSep SEC s2000, 300x7.8 mm, Phenomenex). In the case of insufficient RCP (<90%), the protein was purified using a Bio-Spin 6 desalting column (Bio-Rad) according to the manufacturer's instructionsto achieve >95% RCP.

### Serum stability

Murine blood was obtained from the retrobulbar vein of C57BL/6 mice and collected in 1.5 mL Eppendorf tubes. The blood was kept at room temperature for 2 h and centrifuged at 12.000 rpm for 15 min. The serum was collected as supernatant and incubated with the radiolabeled ^89^Zr-hCD4-Mb. HPLC measurements were performed at 0, 24, and 72h after tracer incubation and the % of ^89^Zr-hCD4-Mb and ^89^Zr-DTPA was determined.

### CD4^+^ cell isolation and culture

Murine CD4^+^ cells were isolated from the lymph nodes and spleens of female C57BL/6 mice using CD4^+^ magnetic microbeads (Miltenyi Biotec). Six-well plates were precoated at +4°C overnight with 0.5 µg/ml αCD3 and 5 µg/ml αCD28 mAbs (Bioxcell) in 5 ml/well PBS. The freshly isolated CD4^+^ cells were cultured in RPMI medium (Lonza Biosciences) supplemented with 10% FCS, 0.5% penicillin/streptomycin (P/S), 0.5 µM β-mercaptoethanol and 1% Insulin Transferrin Selenium (ITS). After 24 h, 30 U/ml IL-2 and 0.5 ng/ml IL-7 were added to each well. The medium was renewed every 2 to 3 days, and the cells were cultured for no longer than 14 days.

Human CD4^+^ cells were isolated from whole-blood samples of healthy donors using StraightFrom Whole Blood CD4 MicroBeads (Miltenyi Biotec) immediately before each *in vitro* experiment.

### Tumor cells

The hCD4^+^ hematopoietic peripheral blood acute lymphoblastic leukemia tumor (HPB-ALL) and hCD4^-^ diffuse histiocytic lymphoma (DHL) cell lines were purchased from the German Collection of Microorganisms and Cell Cultures (DSMZ) and cultured in RPMI-1640 medium, supplemented with 10% Fetal Calf Serum (FCS) and 1% P/S. The MC38 murine colon adenocarcinoma cell line was purchased from Kerafast and cultured in DMEM (Lonza Biosciences) supplemented with 10% FCS, 1% P/S, and 1% HEPES (Lonza Biosciences). The B16F10 murine melanoma cell line was purchased from ATCC and cultured in DMEM, supplemented with 10% FCS and 1% P/S. The S2WTP3 (PyMT) triple-negative breast cancer cell line was kindly provided by Andreas Moeller (Queensland University, Australia) and cultured in DMEM supplemented with 10% FCS, 1% P/S, and 1% pyruvate (Sigma).

### *In vitro* binding assays

The *in vitro* binding of ^89^Zr-mCD4-Mb or ^89^Zr-hCD4-Mb was assessed using freshly isolated or immortalized murine or human CD4^+^ cells. Different numbers of cells were incubated with 10 ng of ^89^Zr-mCD4-Mb or ^89^Zr-hCD4-Mb for 90 min. The cells were washed with PBS/2% FCS and resuspended in 200 µL PBS / 2% FCS. For the antigen-blocking experiments, a 100-fold excess of unlabeled CD4-Mb was added to the wells 30 min before tracer incubation. The residual cell-bound radioactivity was measured in a γ-counter (Wallac 1480 WIZARD 3” Gamma Counter; PerkinElmer). The immunoreactive fraction of each radioimmunoconjugate was determined by calculating the maximum specific binding (Bmax) of a one-site nonlinear regression model within an antigen excess assay with an increasing number of cells (0 - 32 x 10^6^).

### Animals

All experiments were performed according to the animal use and care protocols of the German Animal Protection Law and were approved by the Regierungspräsidium Tübingen. 6-10-week-old NOD SCID gamma (NSG, NOD.Cg-*Prkdc^scid^ Il2rg^tm1WjI^*/SzJ, Charles River Laboratories), C57BL/6 (Charles River Laboratories), and human CD4-Knock-in (hCD4-KI, C57BL/6J-Cd4^tm1.1(CD4)Geno^, Genoway, in-house breeding) mice were bred under specific pathogen-free conditions with free access to food and water *ad libitum*.

### Tumor models and immunotherapies

10 x10^6^ HPB-ALL or DHL cells were injected *s.c.* in the right flank of NSG mice in 200 µl of 50% PBS/50% Matrigel (Corning)*.* 0.125 x10^6^ MC38 or B16F10 tumor cells were injected *s.c.* into C57BL/6 mice in 50% PBS/50% Matrigel (Corning). A total of 0.5 x10^6^ PyMT mammary tumor cells were orthotopically injected into the 4^th^ mammary fat pad in PBS into C57BL/6 or hCD4-KI mice.

C57BL/6 and hCD4-KI mice with PyMT tumors were injected *intraperitoneally* (*i.p.*) with 200 µg of αPD-1 (clone: RMP1-14l) and 50 µg of α4-1BB (clone: 3H3). C57BL/6 mice with MC38 tumors were injected *i.p.* with 500 µg of αPD-L1 (10F.9G2) and αLag-3 (C9B7W) or isotype control mAbs (LTF-2 and HRPN, respectively). All therapeutic antibodies were purchased from Bioxcell.

### Positron Emission Tomography (PET) and Magnetic Resonance Imaging (MRI)

For simultaneous PET/MRI, experimental mice received 2 MBq/10 µg of ^89^Zr-mCD4-Mb or ^89^Zr-mCD4-Mb via tail vein injection. Static scans (600 s) were acquired under 1.5% isoflurane anesthesia (100% oxygen) with an in-house manufactured PET insert 80 at 6, 24, and 48 h post tracer injection. Simultaneous T2-weighted MR images [repetition time (TR): 1800 ms, echo time (TE): 4.763 ms, field of view (FoV) 76.8 × 34.8 × 22.8, × mm^3^, matrix 256 × 256 × 64, resolution 0.25 × 0.25 × 0.25 μm^3^] were acquired in a 7T small animal MR system (ClinScan; Bruker BioSpin).

### Image analysis

PET images from list mode were reconstructed using 2-dimensional ordered subset expectation maximization (OSEM-2D), applied to a Gaussian filter of 1.5 mm, and registered to the anatomical T2 MR images using Inveon Research Workplace (Siemens Preclinical Solutions). Volumes of interest (VOIs) of the organs of interest were created based on the anatomical MR images. The uptake values of the respective organs (%ID/mL) were calculated based on the Bq/mL after correction for radioactive decay and normalization to the injected activity. For visual comparison between the PET images, the signal intensity between the groups and the color scale was normalized within one experiment.

### *Ex vivo* biodistribution

The experimental mice were sacrificed by cervical dislocation under deep anesthesia after the final imaging time point. Organs were harvested, and radioactivity was measured by γ-counting using an energy window between 350 and 650 keV. Standardized aliquots of the injected tracer were added to the wells for quantification. The values for each organ are expressed as the percentage of the overall injected dose per g (%ID/g), corrected for radioactive decay, and normalized to the injected activity.

### Immunohistochemistry

After γ-counting of the organs, the tumors, spleens, and lymph nodes were fixed in formalin and embedded in paraffin. For histology, 3-5 µm-thick sections were cut and stained with hematoxylin and eosin (H&E). Immunohistochemistry was performed on an automated immunostainer (Ventana Medical Systems, Inc.) according to the manufacturer's protocols for open procedures, with slight modifications. All slides were stained with antibodies against CD4 (SP35; Zytomed, Berlin, Germany) and CD3 (SP7; DCS Innovative Diagnostics-Systeme GmbH & Co., KG). Appropriate positive and negative controls were used to confirm the adequacy of the staining. All samples were scanned with a Ventana DP200 (Roche, Basel, Switzerland) and processed with the Image Viewer MFC Application. Final image preparation was performed with Adobe Photoshop CS6.

### Immunofluorescence

Paraffin-embedded MC38 tumor tissues were cut into 3-5 µm-thick sections, deparaffinized, unmasked with EDTA buffer (pH 9.0; Thermo Fisher Scientific), and washed with distilled water, PBS (Sigma´-Aldrich), and PBS containing BSA (Aurion) and Tween 20 (Roth). The tissue sections were blocked with donkey serum, incubated with primary antibodies against CD3 (DCS) and CD4 (R&D Systems), and visualized by incubation with Alexa Fluor 488 donkey anti-rabbit (Dianova), Cy3 donkey anti-goat (Dianova) and Cy3 donkey anti-human (Dianova) antibodies. Nuclei were stained with DAPI (Sigma-Aldrich). Images were analyzed using Zeiss LSM 800 and ZEN 2.3 software (blue edition). CD4 fluorescence staining and nuclei were quantified using ZEN Module Image Analysis and by manually counting.

### Flow cytometry

Tumors were excised and digested for 30 min at 37°C on a shaker with 23 mg/ml Collagenase (Sigma-Aldrich) and 2.3 mg/ml DNAse (Sigma-Aldrich) in RPMI 2% FCS. Single-cell suspensions were prepared by passing the tumors through 70 µM and 40 µM cell strainer sieves. The cell pellet was incubated at room temperature in ACK lysis buffer (Gibco Life Technologies). The following fluorescent dyes were used for staining: viability ghost dye UV450 (Cytek), BUV395-CD45 (Clone: 30-F11, BD Horizon), BUV805-CD3 (Clone: 17A2, Thermofisher), PerCP-CD4 (Clone: GK1.5, BioLegend), AF700-CD8a (Clone: 53-6.7, BioLegend), BV650-CD69 (Clone: H1.2F3; BioLegend), PE-mOX40 (Clone: OX-86, BioLegend). HPB-ALL and DHL cells were pelleted and stained with the following fluorescence dyes: FITC-hCD4 (Clone: OKT4; Biolegend) and viability ghost dye UV450 (Cytek). Cell suspensions were measured on a Cytek Aurora cytometer. Data were analyzed with FlowJo Software.

### Statistical analyses

All data were analyzed using GraphPad Prism, Version 9 or later (GraphPad Software, Inc., San Diego, California, USA). The values were expressed as the arithmetic mean ± standard error of the mean (SEM), if not otherwise stated in the figure legend. For statistical analyses, unpaired t-tests were applied for pairwise comparisons. Ordinary one-way ANOVA or two-way ANOVA was used for multiple group comparisons and was corrected for multiple comparisons using the Tukey post-hoc test. Adjusted *P* values less than 0.05 were considered significant, and significance levels are indicated as follows: * for ≤0.05, ** for ≤0.01, and *** for ≤0.001.

## Supplementary Material

Supplementary figures.

## Abbreviations

CIT: checkpoint inhibitor therapy
CTL: cytotoxic T lymphocytes
CTLA-4: cytotoxic T lymphocyte antigen 4
dfo: desferrioxamine
DHL: diffuse histiocytic lymphoma
FCS: Fetal Calf Serum
FoV: field of view
H&E: hematoxylin and eosin
hCD4-KI: human CD4^+^ knock-in
HPB-ALL: hematopoietic peripheral blood acute lymphoblastic leukemia tumors
HPSEC: high-performance size exclusion chromatography
ICI: immune checkpoint inhibitor
ID: injected dose
IF: immunofluorescence
IFNγ: Interferon gamma
IHC: immunohistochemistry
IL: Interleukin
Lag-3: lymphocyte-activation gene 3
mAb: monoclonal antibody
Mb: minibody
MRI: magnetic resonance imaging
NK: natural killer
OSEM-2D: 2-dimensional ordered subset expectation maximization
P/S: penicillin/streptomycin
PBS: phosphate buffer saline
PBST: phosphate-buffered saline with tween
PD-L1: programmed death ligand
PET: positron emission tomography
RCP: radiochemical purity
SD: standard deviation
SEM: standard error of the mean
TA: tumor antigen
TE: echo time
Th: T helper
TME: tumor microenvironment
TR: repetition time
VOI: volumes of interest
WT: wild-type
