# Supplementary material for: In-depth cross-validation of human and mouse CD4-specific minibodies for noninvasive PET imaging of CD4+ cells and response prediction to cancer immunotherapy
Source: Theranostics. 2024 Aug 1;14(12):4582–97. doi: 10.7150/thno.95173 (PMC11373626; doi:10.7150/thno.95173)
Supplement: Supplementary file 1 — Supplementary figures. [file thnov14p4582s1.pdf]

# SUPPLEMENTAL FIGURES

## In-depth cross-validation of human and mouse CD4-specific minibodies for noninvasive PET imaging of CD4$^+$ cells and response prediction to cancer immunotherapy

Stefania Pezzana[1], Simone Blaess[1], Jule Kortendieck[1], Nicole Hemmer[1], Bredi Tako[1,2], Claudia Pietura[1], Lara Ruoff[1], Simon Riel[7], Martin Schaller[7], Irene Gonzalez-Menendez[3,4], Leticia Quintanilla-Martinez[3,4], Alessandro Mascioni[5], Argin Aivazian[5], Ian Wilson[5], Andreas Maurer[1,3], Bernd Pichler[1,3,6], Manfred Kneilling[1,3,7], Dominik Sonanini[1,3,8,*]

[1] Werner Siemens Imaging Center, Department of Preclinical Imaging and Radiopharmacy, University Hospital Tuebingen, Eberhard Karls University, Tuebingen, Germany

[2] Department of Nuclear Medicine, University Hospital Tuebingen, Eberhard Karls University, Tuebingen, Germany

[3] University of Tuebingen, Cluster of Excellence iFIT (EXC2180) "Image-Guided and Functionally Instructed Tumor Therapies", Tuebingen, Germany

[4] Department of Pathology and Neuropathology, Eberhard Karls University, Tuebingen, Germany

[5] ImaginAb, Inglewood, United States of America

[6] German Cancer Consortium (DKTK) and German Cancer Research Center (DKFZ) partner site Tuebingen, Tuebingen, Germany

[7] Department of Dermatology, University Hospital Tuebingen, Eberhard Karls University, Tuebingen, Germany

[8] Department of Medical Oncology and Pneumology, University Hospital Tuebingen, Eberhard Karls University, Tuebingen, Germany

* Corresponding author

Correspondence to: Dominik Sonanini

Department of Preclinical Imaging and Radiopharmacy

Eberhard Karls University Tübingen

Röntgenweg 13, 72076 Tübingen, Germany

Phone: +49-7071-29-87443

Fax: +49-7071-29-4451

E-mail: Dominik.Sonanini@med.uni-tuebingen.de

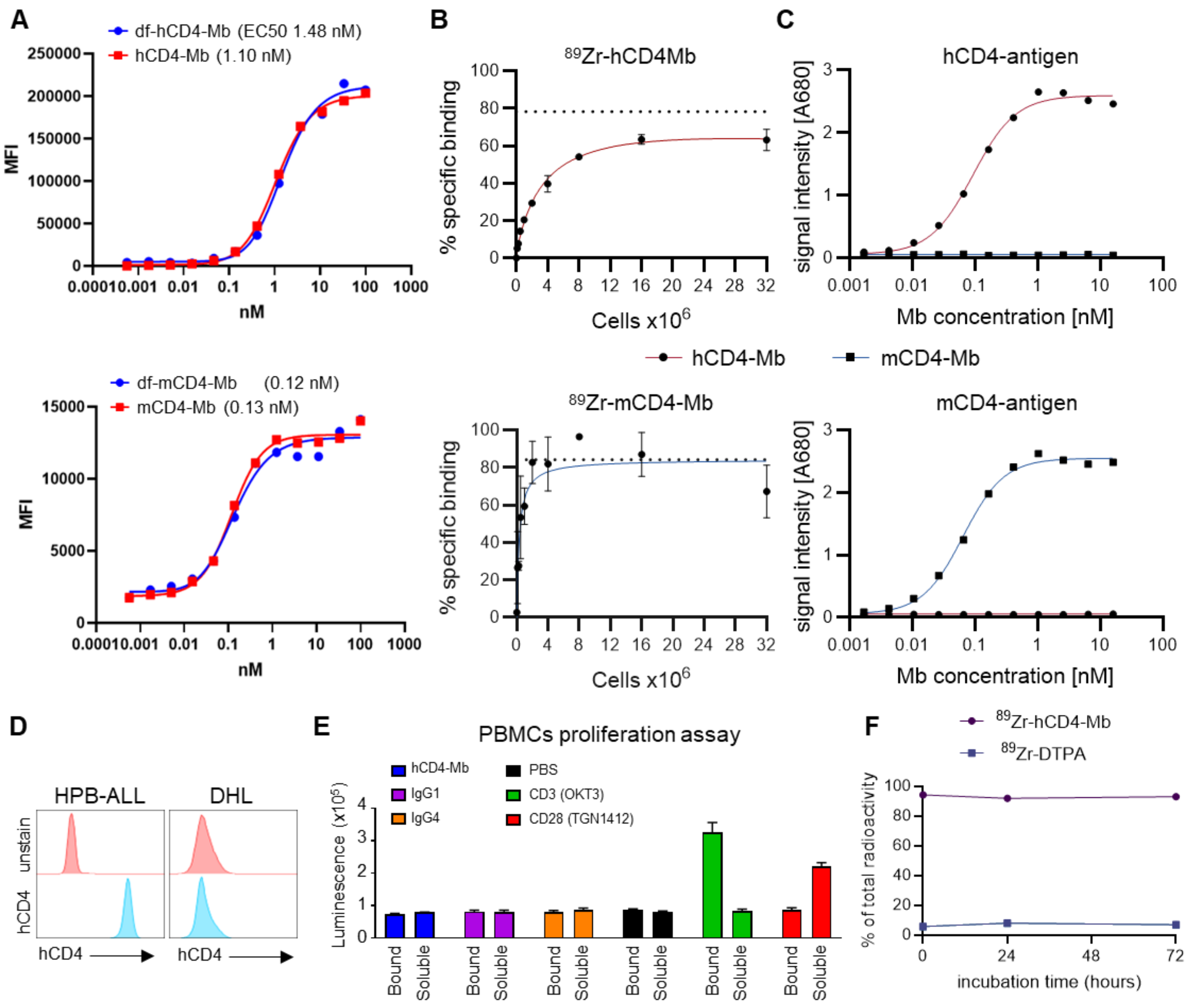


**FIGURE S1** ***In vitro*** **validation of $^{89}$Zr-h/mCD4-Mbs.** (A) Flow cytometry to determine the EC50 of h/mCD4-Mbs with and without dfo conjugation on hCD4 or mCD4-transfected NIH-3T3 cells. (B) Maximum binding ($B_{max}$) assay with 10 ng of $^{89}$Zr-h/mCD4-Mb per well and increasing numbers of hCD4$^+$ HPB-ALL cells or freshly isolated mCD4$^+$ cells (triplicates). $B_{max}$ was calculated by using a one-site specific nonlinear fitness model. For $^{89}$Zr-mCD4-Mb, two values were excluded from the analysis. (C) ELISA of human CD4 (hCD4)-antigen and mouse CD4 (mCD4)-antigen with an increasing number of $^{89}$Zr-h/mCD4-Mbs (D) Flow cytometry of hCD4 expression on HPB-ALL and DHL cell lines. (E) PBMC proliferation assay after plate coating or the addition of soluble antibodies (hCD4-Mb, IgG1, IgG4, CD3, or CD28) for 5 days. (F) % of total radioactivity measured by HPLC of $^{89}$Zr-hCD4-Mb and free $^{89}$Zr-DTPA at 0, 24, and 72 h after 89Zr-hCD4-Mb incubation in mouse serum. The data are presented as mean ± SD.

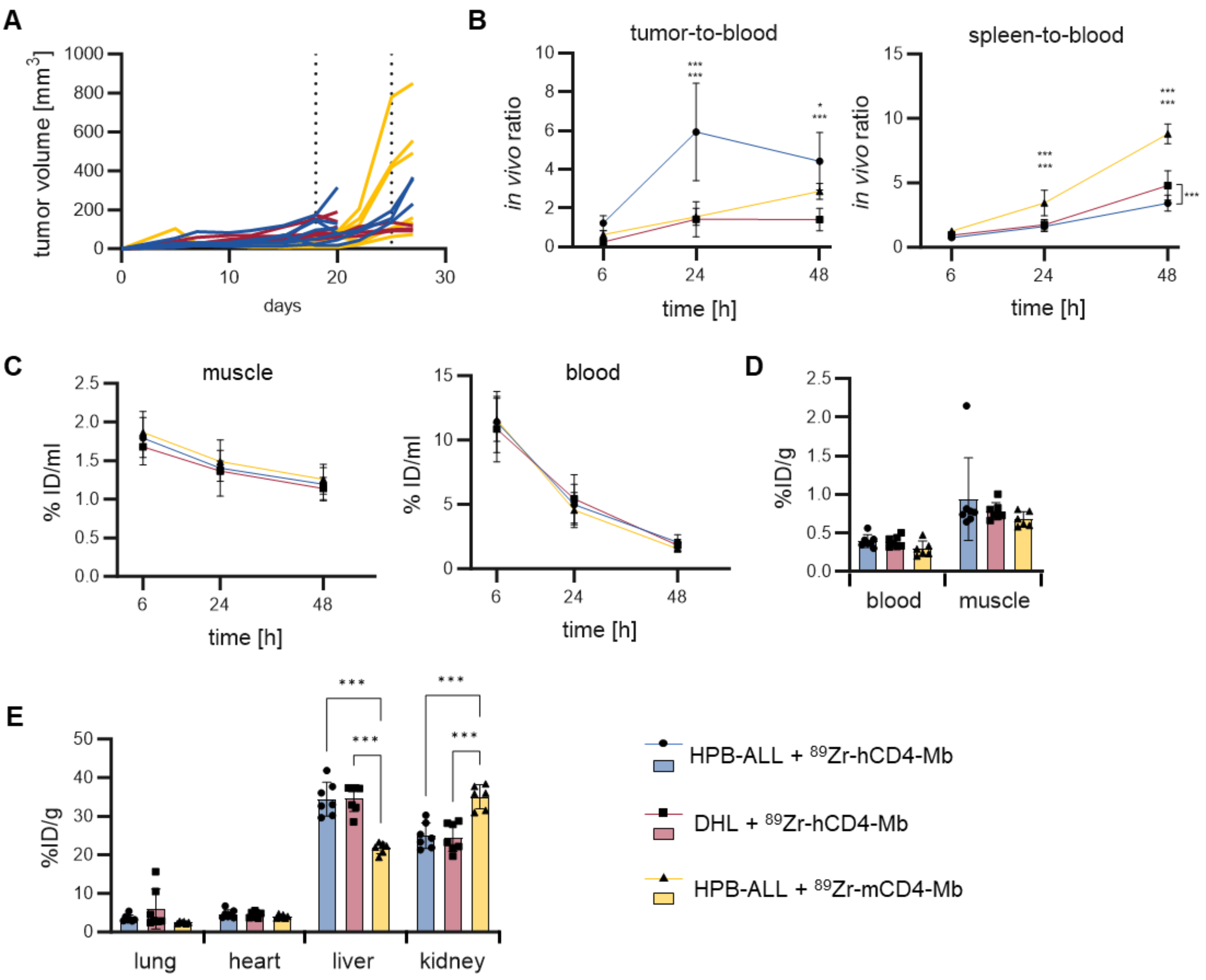


**FIGURE S2** ***In vivo*** **$^{89}$Zr-hCD4-Mb and $^{89}$Zr-mCD4-Mb binding to CD4$^{+}$ cells in immunodeficient NSG mice.** (A) Tumor growth of subcutaneously inoculated (hCD4$^{+}$) HPB-ALL and (hCD4$^{-}$) DHL tumors. Mice were acquired for PET/MRI when tumors reached a volume between 100 and 300 mm³ (Two separate cohorts, indicated by black dotted lines). (B) Tumor-to-blood and spleen-to-blood *in vivo* $^{89}$Zr-CD4-Mb uptake ratios at 6, 24, and 48 h after tracer injection. (C) *In vivo* $^{89}$Zr-CD4-Mb uptake in muscle and blood at 6, 24, and 48 h after tracer injection. (D,E) *Ex vivo* organ $^{89}$Zr-CD4-Mb uptake 48 h after tracer injection measured by γ-counting. *P* values were calculated by two-way ANOVA (B, C) or ordinary one-way ANOVA (D, E) using Tukey's post-hoc test. **p* < 0.05, ****p* < 0.001.

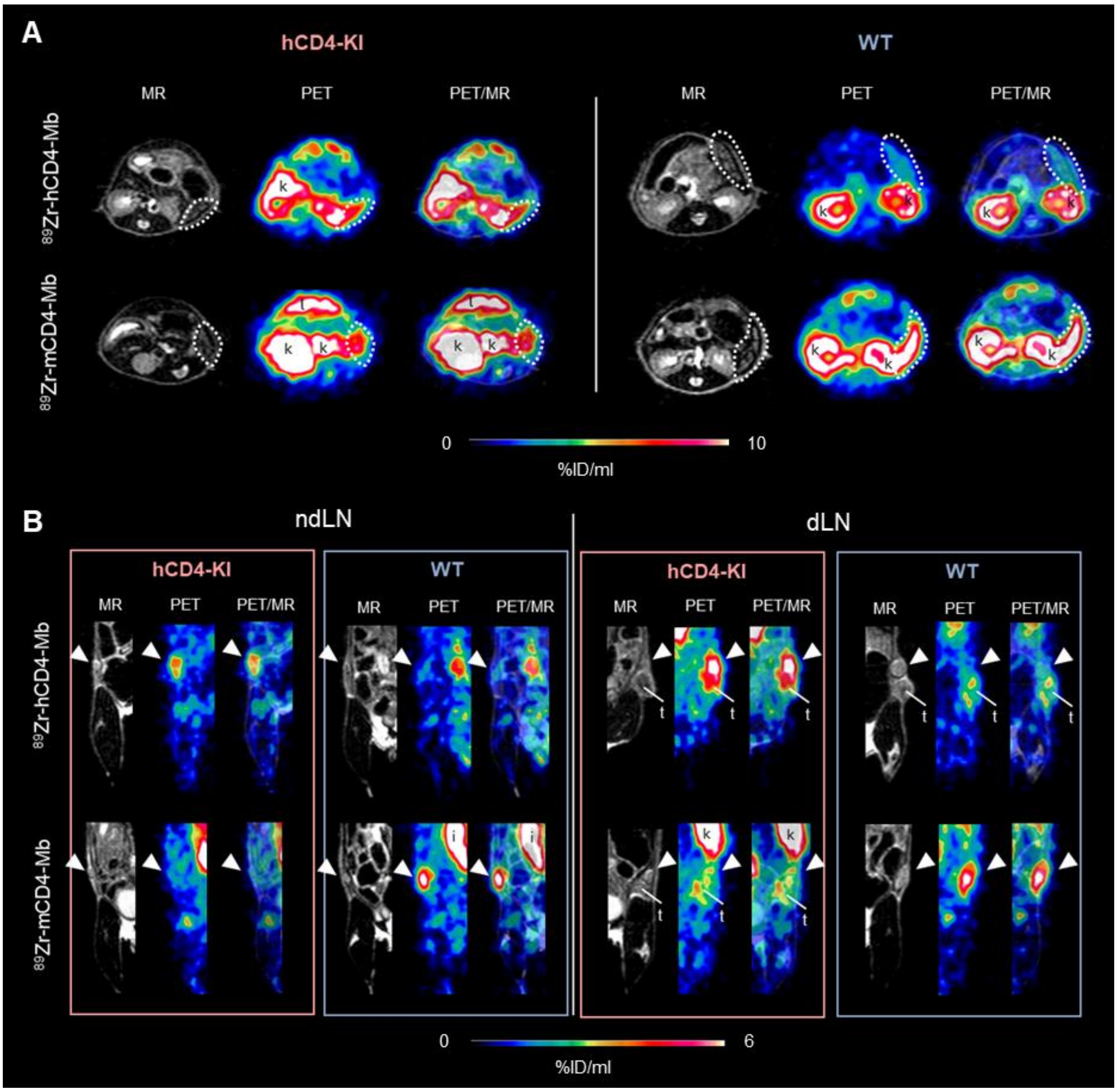


**FIGURE S3 $^{89}$Zr-CD4-Mb immunoPET uptake in lymphatic organs of WT and hCD4-KI PyMT tumor-bearing mice.** (A) Representative MR (left), PET (middle) and PET/MR (right) images of spleen (white-dotted circle) in hCD4-KI mice and WT mice injected with $^{89}$Zr-h/mCD4-Mb. (B) Representative MR (left), PET (center) and PET/MR (right) images of contralateral (non-draining, nd) and tumor-draining lymph nodes (dLN) (white triangle) in hCD4-KI mice and WT mice injected with $^{89}$Zr-h/mCD4-Mb.

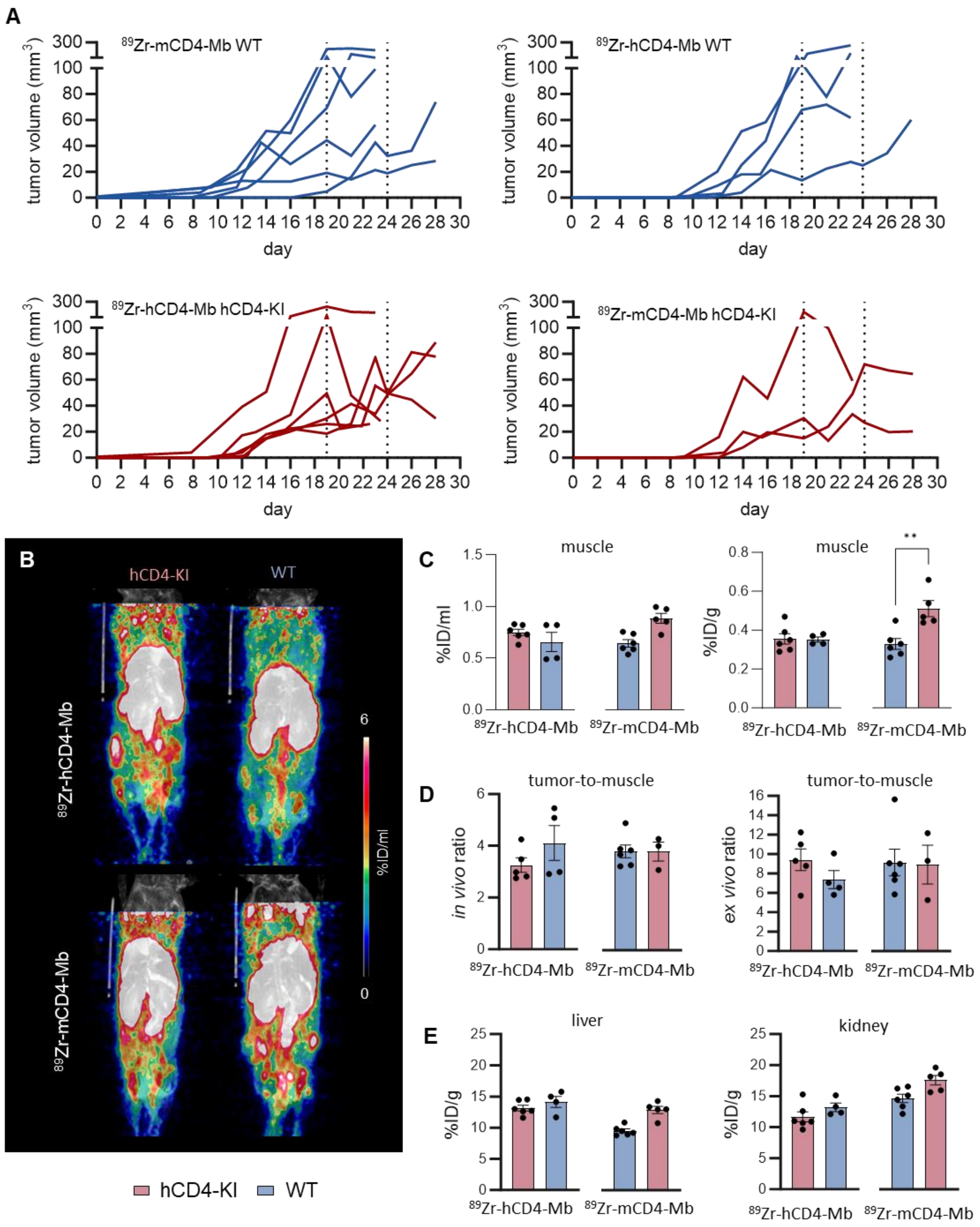


**FIGURE S4 $^{89}$Zr-CD4-Mb immunoPET imaging of WT and hCD4-KI PyMT tumor-bearing mice.** (A) Tumor growth of PyMT tumor-bearing mice. Mice were separated into two cohorts based on the tumor volume in each group at different αPD-1/α4-1BB administration time points (indicated

by black dotted lines). (B) Representative *in vivo* mean intensity projections (MIPs) of $^{89}$Zr-h/mCD4-Mb immunoPET/MRI. *In vivo* and *ex vivo* quantification of $^{89}$Zr-h/mCD4-Mb (C) muscle uptake and (D) the tumor-to-muscle ratio. (E) *Ex vivo* $^{89}$Zr-h/mCD4-Mb uptake quantification in the liver and kidneys. All the data represent the 48 h timepoint after tracer injection. *P* values were calculated by an unpaired t-test. **$p < 0.01$.

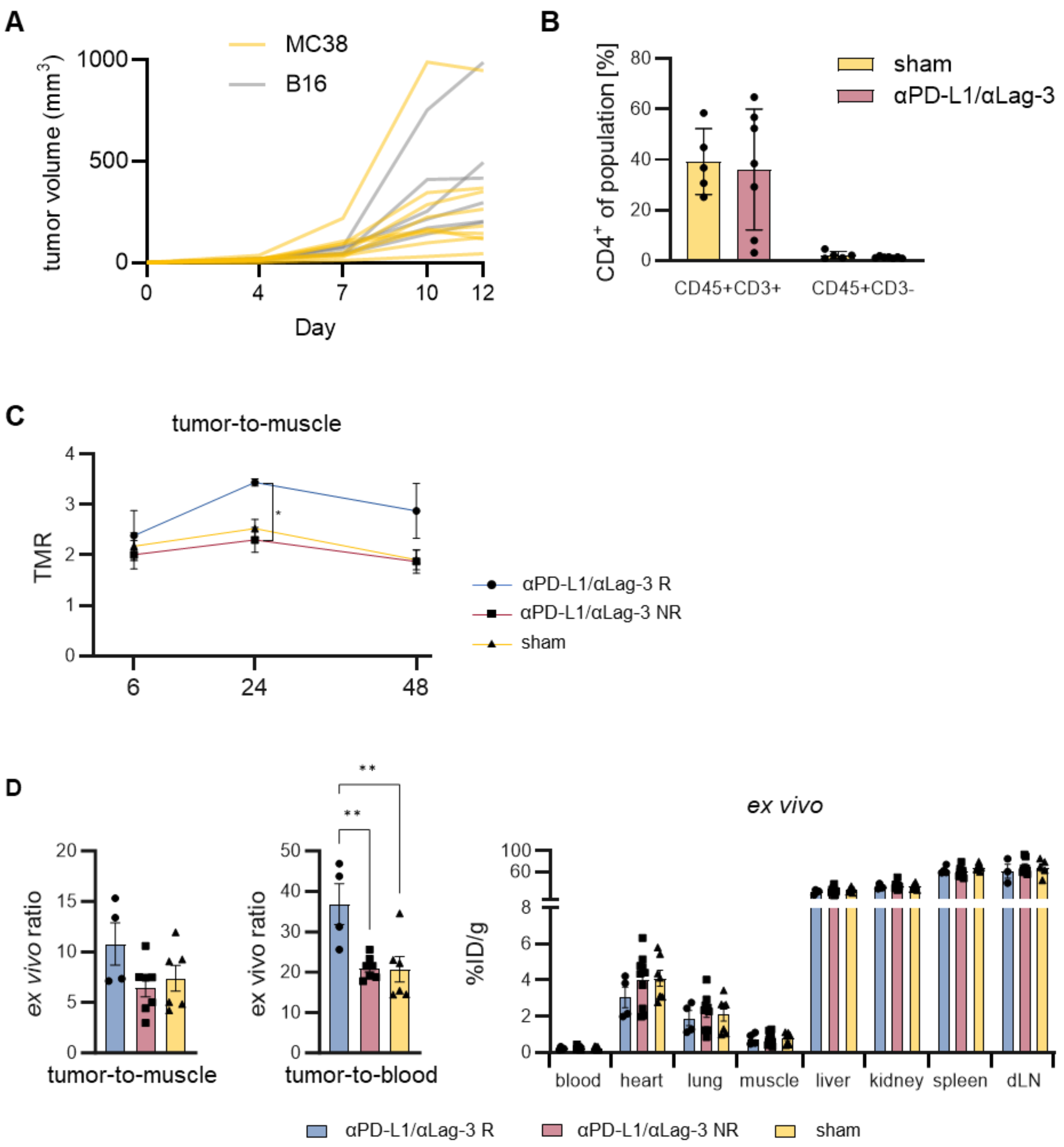


**FIGURE S5 Monitoring of endogenous mCD4⁺ cell infiltrates in response to cancer ICI.** (A) Tumor growth curves of MC38 and B16 tumors. (B) Flow cytometry characterization of CD4-expression of myeloid cell infiltrates in sham-treated or αPD-L1/αLag3-treated MC38 tumors (C) *In vivo* tumor-to-muscle $^{89}$Zr-mCD4-Mb uptake ratio at 6, 24, and 48 h after tracer injection into MC38 tumor-bearing mice treated with αPD-L1/αLag-3 mAbs 7 days before the first PET/MRI timepoint. (D) *Ex vivo* tumor-to-muscle and tumor-to-blood uptake ratios and organ biodistribution 48 h after tracer injection in MC38 tumor-bearing mice treated with αPD-L1/αLag-3 mAbs 7 days prior to the

first PET/MRI timepoint. *P* values were calculated by two-way ANOVA (B) using Tukey's post-hoc test. $^{**}p < 0.01$.
